# The Role of Gluten in Food Products and Dietary Restriction: Exploring the Potential for Restoring Immune Tolerance

**DOI:** 10.3390/foods12224179

**Published:** 2023-11-20

**Authors:** Li Ye, Wenyu Zheng, Xue Li, Wenmin Han, Jialing Shen, Qiuya Lin, Liyan Hou, Lan Liao, Xin’an Zeng

**Affiliations:** 1 Guangdong Key Laboratory of Intelligent Food Manufacturing, Foshan University, Foshan 528225, China; yone1031@163.com (L.Y.); zhengwenyyy@163.com (W.Z.); lixue02022@163.com (X.L.); 18075354234@163.com (W.H.); 18318491242@163.com (J.S.); 15016308592@163.com (Q.L.); a3076439257ccm_1@163.com (L.H.); 2 Department of Food Science, Foshan University, Foshan 528000, China; 3 School of Food Science and Engineering, South China University of Technology, Guangzhou 510640, China

**Keywords:** gluten, food processing, interaction, digestibility, celiac disease, dietary restriction, oral tolerance

## Abstract

Wheat is extensively utilized in various processed foods due to unique proteins forming from the gluten network. The gluten network in food undergoes morphological and molecular structural changes during food processing, affecting the final quality and digestibility of the food. The present review introduces the formation of the gluten network and the role of gluten in the key steps of the production of several typical food products such as bread, pasta, and beer. Also, it summarizes the factors that affect the digestibility of gluten, considering that different processing conditions probably affect its structure and properties, contributing to an in-depth understanding of the digestion of gluten by the human body under various circumstances. Nevertheless, consumption of gluten protein may lead to the development of celiac disease (CD). The best way is theoretically proposed to prevent and treat CD by the inducement of oral tolerance, an immune non-response system formed by the interaction of oral food antigens with the intestinal immune system. This review proposes the restoration of oral tolerance in CD patients through adjunctive dietary therapy via gluten-encapsulated/modified dietary polyphenols. It will reduce the dietary restriction of gluten and help patients achieve a comprehensive dietary intake by better understanding the interactions between gluten and food-derived active products like polyphenols.

## 1. Introduction

**Figure 1:**
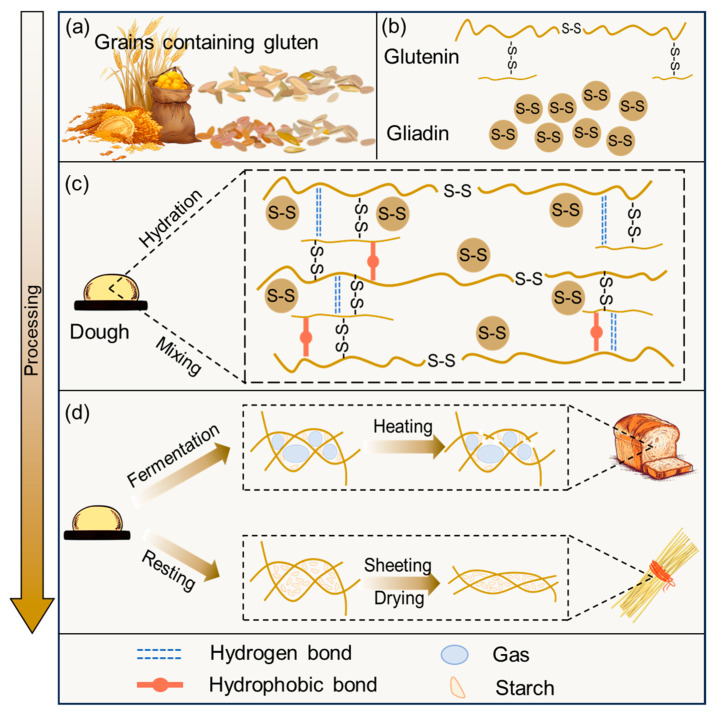
Classification and function of gluten. (**a**) Grains consist of gluten. (**b**) Structure of glutelin and gliadin. (**c**) Gluten structure of the dough. (**d**) Changes of gluten in the dough after processing.

Cereals are a paramount category among global crops, playing a pivotal role in sustaining human nutrition. While cereals exhibit a lower protein content than legumes, their total annual grain production is much higher than legumes. This prodigious output renders cereals formidable contributors to the global protein supply, approximately tripling the protein provision compared to legumes [1]. Research on cereal proteins dates back to more than 200 years ago. The gluten in wheat, which was initially identified by Jacopo Bartolomeo Beccar in Bologna, Italy, in 1728, is of utmost concern [2]. It was initially defined as the rubbery substance retained when washing wheat dough to remove starch granules and water-soluble components [3]. The biological definition usually derives from “storage proteins of the wheat grain”. This protein has also been given different names in other different grains: hordeins in barley, secalin in rye, and avenins in oats [4] (Figure 1a). Gluten is a complex mixture of proteins, including water-insoluble glutenin and alcohol-soluble gliadin [5]. It is characterized by repetitive sequences rich in glutamine and proline. Gluten proteins interact, creating a viscoelastic gluten network that can be processed into various food products. The gluten network is many widely consumed foods’ main structural macro-construct component. Its unique viscoelasticity, gas retention, and cohesiveness are crucial to the quality of cereal-based products, such as bread, cakes, biscuits, and pasta [6]. Furthermore, food processing induces alterations in the structure of gluten, thereby directly altering the sensitivity of gluten to digestion [7]. In particular, various processing methods generally result in the deformation and opening of gluten, increasing its susceptibility to enzymatic degradation by digestive enzymes and thus improving gluten digestibility [8]. However, many studies have found that roasting appears to cause gluten aggregation, reducing gluten digestibility [9]. Research on the digestibility of gluten under different factors provides a reference point for the control of gluten processing conditions to achieve a well-rounded diet for the population.

It causes an adverse immune response in some people, despite the widespread use of gluten in foods. In particular, gluten has more and more processing and application methods in modern society. It may induce interactions between gluten and other components in the food, thereby increasing the likelihood of consumer exposure to wheat-sensitive components [10]. The most common disorder is celiac disease, an autoimmune condition of the small intestine triggered by the consumption of gluten, in which patients exhibit symptoms, including diarrhea, which is frequently accompanied by other gastrointestinal issues and signs of malnutrition [11]. A gluten-free diet has been the most effective treatment for CD due to its efficacy and non-toxicity. However, the widespread implementation of gluten-free diets without medical advice will increase the burden on consumers. People with CD need a healthy and long-lasting effective way to reduce gluten dietary restrictions. The loss of oral tolerance causes the persistent deterioration of the intestine in CD patients [12]. However, studies reported that it could restore immune tolerance by regulating the intake dose or modifying refined gliadins (main antigenic subunits of gluten) [13]. This tolerant mechanism is directly related to the formation and functional presentation of tolerogenic dendritic cells (tolDCs) and regulatory T cells (Treg). Some intervention substances or methods that reverse the persistent pro-inflammatory environment caused by ingesting gluten are vital to the recovery of oral tolerance. To do so, dietary polyphenols, a probiotic substance, may be discovered as an effective adjuvant with excellent effects in repairing damaged epithelial cells, inhibiting immature dendritic cells’ (iDCs) maturation and promoting immature T cells to express forkhead box protein P3 (Foxp3). It provides a potential way to achieve oral tolerance. Simultaneously, it also contributes to the advantages of this strategy by way of the properties of gluten and the interaction between gluten and dietary polyphenols for protecting the activity of dietary polyphenols.

In this review, the role of gluten was preliminarily summarized in the key production steps of several common foods. Subsequently, digestibility was discussed under various influencing factors, providing a scientific basis for individuals who need to choose food more carefully due to dietary restrictions or allergies. On this basis, the feasibility of assisted diet therapy, such as polyphenol intervention, was further emphasized. Notably, the design of gluten-encapsulated/modified dietary polyphenols emerged as a pivotal aspect in mitigating the dietary restrictions associated with gluten and reinstating oral tolerance.

## 2. Gluten

### 2.1. Classification of Gluten

Gluten is one of the main protein components in wheat flour, accounting for 80~85% of the total protein. It is characterized by high levels of glutamine, proline, and hydrophobic amino acids [14]. Gluten is classified into three groups: sulfur-rich, sulfur-poor, and high molecular weight. The proteins in each group have similar structures, including signal peptides, non-repeating N-terminal regions, non-repeating C-terminal regions, and long repetitive center regions. Sulfur-rich gluten forms intrachain disulfide (SS) bonds, while sulfur-poor gluten does not [15]. In addition, gluten plays a pivotal role in defining the structural and functional attributes of wheat-based products. According to the solubility in an alcohol solution, it is divided into soluble monomer gliadin and insoluble polymer glutenin (Figure 1b). The distinctive viscoelastic characteristics of gluten are ascribed to the viscous nature of gliadin and the elastic properties of glutenin, contributing distinctly to the overall quality of processed food products [14].

The molecular weight of gliadin is 30~75 kDa. It is further divided into α-, β-, γ-, and ω-gliadins, with the former three (α-, β-, γ-gliadins) dominating and the latter (ω-gliadins) being less prevalent [16]. This distribution exhibits marked variability contingent upon the wheat varieties and growth conditions. The structure of gliadin comprises a concise N-terminal domain, a repetitive central domain, and a non-repetitive C-terminal domain. Within the central repeat domain, the secondary structure of gliadin is predominantly characterized by reverse and β-turn elements, while α-helix and β-sheet structures are observed in the C-terminus. It is generally believed to form a compact spherical structure in the natural state [17]. This structural complexity underscores the delicate interplay between the intrinsic molecular factors associated with the functional and physiological role of glutenin.

According to the relative molecular weight, glutenin is divided into high-relative-molecular-weight glutenin subunits (LMW-GS, 30~40 kDa) and low-relative-molecular-weight glutenin subunits (HMW-GS, 65~90 kDa). LMW-GS is the main type, accounting for 20% of total gluten, and is further subdivided into B-, C-, and D-types. The primary and secondary structures of LMW-GS are similar to those of gliadin. However, the huge difference between the two is that LMW-GS contains intra-chain and inter-chain SS bonds [18]. In addition, HMW-GS is a secondary component of glutenin, accounting for 10%. It is further divided into two different types: x-type and y-type [19]. The x-type and y-type comprise a relatively small N-terminal domain, a constant-size C-terminal domain, and a central domain with a significant length change. The secondary structure of the N-terminal and C-terminal domains is mainly composed of an α-helix, and the central domain forms a β-spiral super-secondary structure. It is generally believed that HMW-GS adopts a rigid rod-like conformation due to the special structure of its central repetitive domain [20]. This detailed classification sheds light on the different molecular compositions and structural complexity inherent in glutenin subunits and plays a key role in determining the functional properties of glutenin.

### 2.2. The Role of Gluten in Food

The dynamic changes of morphology, distribution, and molecular/structural transformation of gluten during the processing of various foods affect the formation, destruction, and recombination of gluten networks, thus affecting the quality of various foods. Individuals more effectively select or prepare foods that meet their dietary needs by understanding the performance of gluten in a variety of foods and simultaneously comprehending the influence of the gluten network on the characteristics of the final products. It also provides scientific evidence and guidance for people with dietary restrictions and allergies to better meet individual dietary needs and achieve dietary safety.

#### 2.2.1. Dough

##### Formation of the Gluten Network

Gluten proteins within wheat products determine the final quality of the food product [21]. Food products that include gluten generally undergo a series of processing steps, including the addition of water and mixing, to form suitable food raw materials. During dough mixing, the three-dimensional gluten network forms, which provides a framing role for the food system during subsequent processing and has a significant impact on the final product (Figure 1c).

The dried gluten protein at room temperature assumes a glassy state. The water content of the gluten is raised by adding water. When the water content reaches 16%, gluten transforms from a glassy state to a rubber state and elastic material. Many properties of it can be explained by this transition [22]. The gluten amide groups are fully hydrated when the water content reaches 35%. As a result, the dough forms a rubbery and viscoelastic substance. Further addition of water over this will result in dilution of gluten proteins rather than further hydration. However, merely adding water does not form a passable dough. Mixing aids gluten hydration by exposing the new dry surface of gluten to interact with water. It provides sufficient mechanical energy input to infiltrate air into the dough [23]. Mixing causes gliadin to interact with glutenin. From molecular level observations, HMW-GS forms an elastic backbone structure linked by disulfide (SS) bonds. LMW-GS is linked to the HMW-GS polymers by covalent bonds and dispersed in the gluten system in a branched form. Gliadin and glutenin are linked together by non-covalent interactions that fill the space around the wheat gluten polymer. The complex protein interactions eventually combine to form a three-dimensional network [24]. Various chemical bonds are involved in the gluten network, the most prominent of which are SS bonds, hydrophobic interaction forces, and hydrogen bonds. The SS bonds act as strong cross-links in the formation of the dough and stabilize its structure. The hydrophobic interaction forces have a relatively small overall effect on the dough, but their energy increases with temperature, thus stabilizing the gluten network during subsequent dough heating. In addition, the unique role of hydrogen bonding in the dough is the interchange that occurs under pressure, causing the gluten to reorientate [25]. Several models have been proposed to describe the gluten network formed after the hydration of gluten. Among them, the “loop and train” model proposed by Belton is widely accepted. The model suggests that the glutamine residues in gluten form hydrogen bonds with the glutamine residues in the adjacent chains under low hydration. Gluten has almost no ordered conformation at this stage. The system is plasticized, forming an “inter-chain” β-sheet structure with an increase in the degree of hydration. After further hydration, the formation of competitive hydrogen bonds between water and glutamine breaks some inter-chain hydrogen bonds and forms a hydrated “loop” region. The balance is achieved between the hydration “loop” region and the hydrogen bond “chain” region, and the ratio depends on the hydration state of the current dough [26]. The addition of water helps to activate gluten proteins, contributing to the formation of the gluten structure and determining the formation of specific secondary structures of gluten [14]. Li et al. found that the β-sheet structure of gluten increased in hydrated flour [27]. Almutawah et al. [28] pointed out the presence of a considerable number of disordered structures in dried gluten. Hydration leads to conformational relaxation and α-helix and β-sheet formation. Subsequently, Robertson et al. [29] found that hydration stages result in the development of extended hydrated chains and the transformation from a β-sheet to β-turn. It is generally believed that a β-sheet is the most stable secondary structure in gluten, which stabilizes the structure of gluten together with intermolecular SS bonds. The structure of gluten induced by an α-helix is more orderly [30]. Hydration promotes the formation of a more ordered secondary structure of gluten. Gliadin and glutenin interact to form a composite structure, by which both change their original conformations to create a sheet-like phase. Therefore, it is generally believed that the gluten network structure is divided into four structural levels: (1) Molecular level—gluten chains interact with non-covalent interactions through physical forces or SS bonds. (2) Secondary structure level—the isolated fibrils are transformed into a continuous phase of sheet-like morphology under certain conditions, including the proportion of protein components, hydration, and other factors, which are regarded as the basic unit of gluten network construction. (3) Third structural level—the arrangement of gluten protein sheets to form a nanoporous ultrastructure is the basis of water absorption, water retention, and expansion characteristics of gluten protein. (4) Fourth structural level—microstructure arrangement leads to various macroscopic morphology and mechanical properties of gluten [31].

##### Rheological Properties of Dough

Dough rheological properties are critical to the success of food processing. They affect the behavior of the dough during processing and thus the quality of the finished product. Gluten plays a decisive role in the rheological properties of the dough. In general, gluten imparts strength and elasticity to the dough when it contains a large proportion of wheat gluten. The “loop and train” model reveals its elastic characteristics, critical for gas retention during the subsequent fermentation process.

In contrast, gliadin does not form a network structure and does not affect the elasticity of the dough, but it enhances the stickiness of the dough. Edwards et al. [32] found that adding glutenin to the dough increased overall strength. In contrast, the addition of extra gliadins affected dough viscosity. The contribution of gluten to the creep viscoelastic properties of the dough depends on the gliadin–glutenin ratio in its source varieties. Gluten-rich gluten significantly strengthens the dough network and thus enhances the strength of the dough. In contrast, gliadin cannot form a network structure but enhances the dough’s viscous flow properties.

Relevant findings were similarly reported by Sissons et al. [33]. The mixing time significantly affects both the rheological and structural properties of the dough. Insufficient mixing of dough leads to uneven and insufficient distribution of the gluten network. Excessive mixing is not conducive to the development of a dough network, and reduces the uniformity of the spatial distribution of components, making the dough stickier [34,35]. Jia et al. [36] found that sufficient mixing led to an increase in ionic bonds and hydrogen bonds in the gluten. In contrast, excessive mixing leads to more exposure of free thiols. Sluková et al. [37] pointed out that the sulfhydryl (SH) groups of the protein were oxidized to form SS bonds during the mixing process, thereby stabilizing the gluten network structure. The type and degree of change that the dough undergoes during each production step directly shape the quality of the final food product. The occurrence of hydration and mixing during dough formation is critical. The rheological response of the dough to these processes directly determines the food’s production characteristics during processing [38].

#### 2.2.2. Bread

The preparation process of bread involves the dough’s fermentation and heating process steps, which have an important and direct impact on the bread’s sensory quality and nutritional characteristics (Figure 1d). The gluten network plays a crucial role in dough behavior and bread quality by providing viscoelasticity to the dough and promoting air retention during fermentation [39]. The fermentation of dough constitutes a series of intricate biochemical processes. Microbial-induced protein degradation has been noted for its substantial impact on the rheological attributes and comprehensive quality of gluten [40,41,42]. The viscosity, adhesion work, and cohesion of fermented dough increased significantly with the extended fermentation time. In addition, its tensile resistance and ductility decreased, and the fluidity of the dough became worse, showing low elasticity and more plastic deformation behavior [43]. Wang et al. [44] reported that the α-helix content within glutenin macromolecular aggregates diminished, concurrent with an escalation in the β-turn and random coil content throughout the fermentation process. It indicates an augmentation in the flexibility of glutenin macromolecular aggregates in the fermented dough, with some glutenin undergoing hydrolysis into peptides and free amino acids. Simultaneously, Nutter et al. [45] observed varying degrees of depolymerization in gluten, leading to the formation of a fibril network and lamellar structure. This transformation was correlated with an augmentation in the β-sheet structure. From the point of view of the secondary structure and rheological properties, it appears that fermentation destroys some of the superior properties of the dough. However, the dough expands during fermentation to retain gases. The yeast produces CO_2_ in the aqueous phase of the dough, the gluten network acts as an elastic backbone to support the dough, and the viscoelastic dough flows to balance the pressure generated by the additional gas [46]. Excessive pressure within the gas chambers causes the dough to expand and affects the fluffiness of the bread. The diffusion rate of small molecules is significantly influenced by the viscosity of the solution. The highly hydrated gluten matrix that forms a continuous phase in the dough is an elastic material with high apparent viscosity. This characteristic slows down the diffusion rate of CO_2_ through the gluten matrix. Concurrently, the augmentation of the surface area in the liquid film between gas chambers serves to uphold the integrity of the gas chamber, actively facilitating gas retention. Subsequent heating contributes to the production of bread that is not only light but also consistently uniform [47].

Significant macroscopic transformations occur during the bread heating process, as the air chamber nestled between gluten components expands under the influence of heat. A continuous network of gluten acts as the backbone of the dough, conferring it the ability to hold air during the heating phase. However, these air chambers rupture over time to form a macroscopically visible network of pores, giving the distinctive and visible fluffy structure observed in bread. From the perspective of the molecular structure, gluten did not change irreversibly at 20~40 °C [48]. However, the hydrogen bonds in the gluten were broken when the heating temperature reached about 45 °C, and the conformation changed, resulting in the exposure of hydrophobic groups. The hydrophobic interaction is enhanced as the temperature increases, exposing a large number of hydrophobic groups that cause protein aggregation [49]. When the temperature was increased to 90 °C, the molecular weight of glutenin was increased by SH-SS exchange and SH oxidation. The gliadin actively participates in the formation of a gluten network at temperatures exceeding 90 °C by means of the SH-SS exchange reaction, thereby enhancing intramolecular cross-linking within the gluten structure [3,50]. In addition, wheat starch is also an important ingredient in wheat flour [51]. The gluten network interacts with substances such as starch in the dough system and facilitates the aggregation and formation of aggregates. Starch fills the voids in the gluten network and plays a role in dough stability after heating and pasting [52]. Further research into the theoretical modeling of gluten interactions with other components is desirable to understand the role of the gluten network in bread [53].

#### 2.2.3. Noodles

The production of noodles diverges from that of fermented foods like bread. The main influence of gluten on product quality is reflected in the resting, extrusion, and drying steps (Figure 1d). The gluten of the mixed dough undergoes stretching and relaxation during the resting process, resulting in the re-aggregation of the gluten network. Liu et al. [54] indicated that the dough resting for more than 30 min would significantly increase the content of tightly bound water in the dough. At the same time, the resting process contributes to the partial restoration of the disrupted SS bonds during mixing, resulting in a significant increase in the content of glutenin macro polymers in the dough [55]. Static resting significantly promotes a uniform and compact distribution of the gluten network, enhancing the texture and cooking characteristics of noodles [56].

During extrusion, the rupture of small air bubbles in the gluten network affects the structure of the air chambers. It reduces the elasticity of the dough, as well as causing damage to the wheat gluten macromolecules. Zhang et al. [55] observed the conversion of β-turn and α-helix structures into β-sheet formations during gluten extrusion. Proper extrusion facilitated the orderly arrangement of gluten fibrils and enhanced the closer cross-linking of gluten, forming a strengthened gluten network. The development of this more robust gluten network contributes to increased elasticity in noodles [57]. The gluten forms a structured gluten network that determines the properties of the pasta during the molding and drying process. Fresh noodles showcase viscoelasticity and a soft texture, while dried noodles acquire a firmer consistency [58]. Lin et al. [59] reported that the alteration in the protein secondary structure during gluten drying primarily involved an increase in β-sheet formation, accompanied by an increase in gauche–gauche–gauche content in the tertiary structure. These changes contributed to the enhancement of the viscoelastic properties of the gluten network. The decrease in SH bond content in gluten increased the aggregation degree of gluten, thereby increasing the cross-linking of gluten to achieve the stability of the gluten network and produce tough, chewy, and elastic noodles [59].

#### 2.2.4. Beer

Beer is a mixture of complex components, and gluten significantly affects the quality of beer. Although gluten is not entirely innocuous, it has the potential to impact the stability of beer. It is well known that the haze and foam stability of beer are important indicators to measure its quality. The colloidal stability of beer is typically attained by eliminating larger particles, particularly flocs formed by the condensation of polyphenols and gluten compounds, commonly referred to as “Chill haze” in clarified beer at lower temperatures [60]. Among these interactions, the extent of haze formation activity is positively correlated with the proline content in glutens binding to polyphenols. Polyphenols responsible for haze exhibit a minimum of two binding sites capable of interacting with glutens. It enables them to form cross-links between proteins, resulting in the generation of insoluble particles that scatter light [61]. The gluten in barley malt undergoes hydrolysis and is sometimes transformed into peptides or amino acids during the beer brewing process. While the majority of gluten in the wort precipitates and is removed with the lees [62], certain glutens, particularly gluten-derived peptides, may persist throughout the brewing process, potentially impacting the gluten-free quality of the beer [63]. However, glutens and their derived peptides also serve as foam stabilizers in beer. The primary structural component of the foam is the protein in beer, imparting excellent stability to the foam. It forms a robust, flexible, and adhesive film that reduces air permeability, inhibiting coalescence and disproportionation [64]. Although gluten-derived peptides have an excellent ability to enter the foam, they are slightly less stable than albumin in terms of foam stability. Therefore, the foam stability in beer is largely dependent on the relative levels of albumin and barley gluten-derived peptides. This explanation is helpful in understanding why over-modified malt may lead to a decrease in beer foam stability [65]. Di Ghionno et al. [66] utilized prolyl endopeptidase to reduce the gluten content in beer and observed that this treatment led to a decrease in foam stability. Considering this complexity, it is of great significance to effectively control the content of gluten and its derived peptides to ensure the quality and safety of beer.

### 2.3. Gluten Digestion

Gluten is a complex protein matrix that undergoes digestion in the human body, considering its bioaccessibility is crucial for providing nutritional advice or designing foods that meet specific nutritional needs. In general, nutrition research typically concentrates on analyzing the effects of food matrices on the release and availability of micronutrients. However, this perspective may not fully apply to gluten. Currently, there is a relative scarcity of information regarding the physical and chemical transformation of glutens in complex food matrices within different gastrointestinal compartments, as well as their biological accessibility [67]. Although research on bioaccessibility is limited, studies on the digestibility of gluten in various food matrices have revealed its bioaccessibility to a certain extent (Table 1). Joye et al. [68] summarized the protein digestibility in cereal products. Both internal and external factors influence the digestibility of protein in cereal foods. Internal factors include the protein amino acid sequence, folding, and cross-linking. The external factors involve the retention of the cell structure and the presence of anti-nutritional factors affecting protein digestion. At the same time, food processing usually aims to improve overall digestibility by affecting these external and internal factors. Common processing methods, such as milling, split the cellular structure, exposing the gluten matrix to the environment and hydrolytic enzymes [68]. Some researchers have recently proposed an emerging processing method for cold atmospheric plasma. It depolymerizes gluten proteins by modifying gluten, reducing the amount of large-sized protein polymers, and decreasing immune reactivity [69,70]. Nevertheless, specific processing methods may eventually result in a decrease in protein digestibility. Xiang et al. [71] reported that microwave heating increases internal cross-linking and the formation of isopeptide bonds within gluten. The cross-links between certain specific amino acids cannot be digested, resulting in a decrease in digestibility. Smith et al. [9] highlighted that the baking process reduces the digestibility of glutens, including those containing sequences that are active against celiac disease. Starch exerts a profound influence on the digestibility of gluten during the production and processing of various cereal foods. Kuang et al. [72] reported that starch significantly enhances the digestion of gluten, inducing changes in the protein configuration and aggregation behavior of glutens. Morphological studies have shown that starch not only functions as a filler particle in the gluten protein network but also dilutes the gluten matrix and impairs the adhesion and connection of the gluten matrix. At the same time, the increase in starch concentration inhibited the formation of disulfide bonds and hindered the aggregation of gluten [24]. Therefore, it is difficult to accurately predict the digestion process of complex food matrices by relying solely on the digestion of purified protein components [67]. Although there has been a certain degree of research on the digestibility of gluten, further research is needed in complex food matrices, especially concerning the transformation process in different gastrointestinal compartments. It is beneficial to gain a more comprehensive understanding of the behavior of gluten in the human body, and it is also of great significance to improve the bioavailability of gluten and understand its role in different food matrices.

## 3. Gluten Protein Induces Celiac Disease and Restores Oral Tolerance

The specialized structure of gluten forms an extensive and uninterrupted network that serves as an elastic skeleton in the food system, contributing to the distinctive characteristics found in various gluten-containing foods. Processing alters the gluten structure through processes such as protein aggregation and chemical modification [85]. Changes in gluten conformation caused by these physicochemical changes not only affect gluten digestibility but may also enhance antigenic potential. The allergenicity of foods can be modified by altering the binding epitopes of potential allergens. Altering the structure of gluten is likely to result in the elimination/exposure of conformational epitopes. In particular, heat treatment can induce modifications in potential allergens’ physicochemical and immunological characteristics by affecting protein digestibility [86]. This is important to consider for gluten, as its structure contains relatively high concentrations of proline residues and glutamine. However, the gastrointestinal digestive enzymes in the human body lack the specificity of the cleavage site after proline and do not have the proteolytic ability required to cleave certain immunogenic gluten peptides effectively. Glutens rich in proline residues cannot be completely digested and can enter through the intestinal cavity, resulting in adverse immune responses in some people [87].

Among them, the most common disease is celiac disease, an autoimmune disease of the small intestine caused by the intake of gluten. Most patients adhere to a gluten-free diet to reverse immune-mediated minor intestinal injury and fully restore absorption function, but it is tough to exclude gluten from the daily diet [88] completely. There is a potential for gluten to interact with other components of the food, increasing the probability that wheat-sensitive components will touch consumers, especially given the variety of processing techniques employed for gluten-containing cereals in modern society. It is critical to comprehend the etiology of celiac disease and identify methods to alleviate gluten-related dietary restrictions and restore oral tolerance to provide a scientific foundation for food processing and design.

### 3.1. Pathological Mechanisms of CD

**Figure 2:**
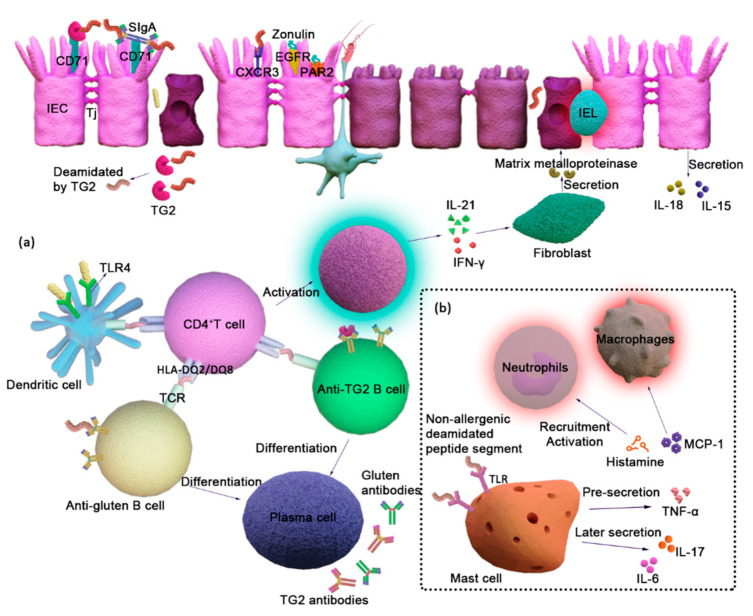
(**a**) Pathological mechanisms of CD. (**b**) Mast cells are involved in the pathophysiology of CD. The allergenic peptides actively enter the lamina propria via the epithelial pathway, where transglutaminase 2 deamidates them, increasing their affinity for HLA-DQ2 or HLA-DQ8, and the antigenic epitopes are picked up by antigen-presenting cells and transmitted to CD4^+^ T cells. B cells use surface receptors to recognize their antigens and to internalize them, and present processed gluten peptides to CD4^+^ cells. When HLA-DQ2 or HLA-DQ8, sensitizing peptides, and various T cell receptors interact, both T cells and B cells are activated. Once activated, gluten-specific CD4^+^ T cells begin to release inflammatory cytokines such as IFN-γ and IL-21, resulting in an inflammatory milieu in the small intestine’s lamina propria. Colonic fibroblasts secrete substantial levels of MMPs (matrix metalloproteinases) after IL-21 and IFN-γ stimulation, and the release and activation of MMP induce extracellular matrix proteolysis. Activated B cells can also develop into plasma cells, which release anti-gluten and TG2 antibodies.

Celiac disease is directly related to the consumption of gluten in wheat, barley, and rye in people of all ages with human leukocyte antigen (HLA) alleles [89]. The pathogenesis of celiac disease is not fully understood, but it mainly occurs in the intestinal duodenum [90]. Gluten is degraded through the oral and gastrointestinal environments to the small intestine, where anti-digestive peptides penetrate the mucosal layer and enter the lamina propria through cross-cellular and intercellular pathways. After entering the lamina propria through the cell barrier, the sensitized peptide is hydrolyzed by TG2 in the lamina propria. The amide bonds of the glutamine side chains break to generate negatively charged glutamate, strengthening the binding of HLA-DQ2/HLA-DQ8 to the antigen presenting cells (APCs). At present, three types of deamidated-allergic peptide presenting cells have been identified: full-time antigen presenting APCs, anti-deamidated gluten B cells, and anti-TG2 B cells [91]. They load the sensitized peptide segments, bind to the CD4^+^ T cell receptor (TCR), activate the T cells to secrete IFN-γ and IL-21, and form a tissue-damaged inflammatory environment [92]. Meanwhile, the B cells, involved in antigen presentation, differentiate into plasma cells, which secrete antibodies of TG2 and deamidated gluten (Figure 2a).

Recent studies have found that mast cells play an essential role in CD by driving innate immune responses. In Figure 2b, non-allergenic peptide segments enter the lamina propria, and TG2 deamidates the peptides, enhancing the recognition ability of mast cells. In the early stages, human tissue-resident mast cells interact with TLR receptors via non-allergenic deamidated peptide segments. This interaction releases pre-synthetic and newly synthesized mediators such as histamine, TNF-α, and MCP-1 to recruit and activate macrophages and neutrophils, thereby participating in early tissue injury. In later stages, when tissue damage expands, mast cells secrete pro-inflammatory factors such as IL-6 and IL-17, maintaining B cells that produce IgA by secreting IL-6 and expressing CD40L. IL-6 and IL-17 also promote local Treg cells to differentiate into Th17 cells, resulting in the failure of oral tolerance [93].

### 3.2. CD-Related Oral Tolerance Mechanisms

The gut immune system is constantly exposed to a diverse mixture of foreign antigens, commensal bacteria, and potential pathogens from the diet. However, the intestinal immune system does not necessarily mount an immune response to non-autoantigens because of the presence of regulatory mechanisms in humans. Understanding the pathogenesis of CD is crucial for effectively avoiding inappropriate responses to innocuous antigens and treating the condition. Oral tolerance is an immune non-response system established by the interaction of oral food antigens with the intestinal immune system. It is mediated by tolDCs, thereby promoting the development of Tregs. As the main effective cells of the tolerance mechanism, they establish most intestinal tolerance mechanisms. In recent years, goblet cells have also been found to play an important role in oral tolerance. These specialized epithelial cells have emerged as key contributors to the immunological processes involved in oral tolerance.

#### 3.2.1. Treg

**Figure 3:**
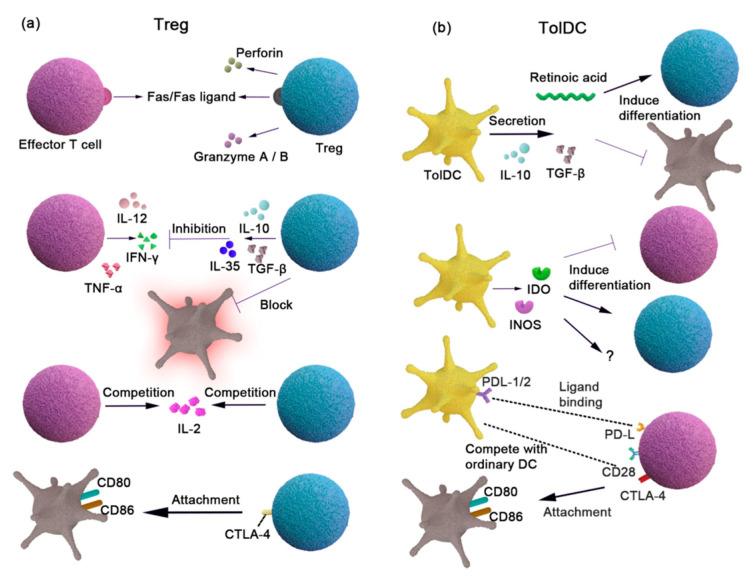
Tolerability mechanism of Treg and tolDC. (**a**) The nTreg mediates cell lysis through granzyme A/B and perforin secreted by cell-to-cell interaction, and iTreg mediates cell lysis through Fas/Fas ligand action on specific CD4^+^ T cells. TGF-β inhibited activated gluten-specific CD4+ T cells and promoted IL-10 secretion. IL-10 inhibits pro-inflammatory factor secretion (such as IFN-γ, IL-12, and TNF-α), blocking wheat toxic protein antigen presentation. This is through the competitive inhibition of IL-2 proliferation to other cells, the upregulation of cAMP, and inhibiting pro-inflammatory gene expression. Treg surface molecular CTLA-4 (CD152) attached to APCs’ stimulus molecules (CD80 and CD86) inhibits intercellular interdependence mechanisms. (**b**) PD-L1/2 (programmed death-ligand 1/2) and CTLA-4 (cytotoxic T-lymphocyte-related protein 4) are highly expressed on the surface and compete with CD80 and CD86 to bind to CD28 on the surface of other DCS. Secretion of IL-10 and TGF-regulatory effects of T cells and Treg; IDO (indoleamine 2, 3-dioxidase) and iNOS (inducible nitric oxide synthase) are secreted to induce Treg.

Treg is critical to human health and the immune system, which prevents inflammatory responses to essential foods while allowing the immune system to target the destruction of pathogens and unwanted antigens. The role of CD-associated Tregs has been described in detail in study [94], and as shown in Figure 3a.

Although thymus-derived pTregs do not appear to be essential in restoring tolerance mechanisms, pTregs play an important regulatory role in oral tolerance. Studies have shown that oral tolerance is able to develop even in the absence of tTreg [95], which further confirms the importance of pTreg. Different pTreg subsets, such as FoxP3^+^ Tregs, LAP Tregs, and Tr1, play important roles in the development of oral tolerance. Studies of villous atrophic small intestinal biopsy specimens have identified the increased Foxp3^+^ expression and increased numbers of CD4^+^CD25^+^Foxp3^+^T cells. It may be related to the upregulation of Foxp3 transcription leading to persistent inflammation [96]. Other Treg subsets have also been described in oral tolerance studies. Oral administration of CD3-specific antibodies induces CD4^+^CD25-LAP^+^ regulatory T cells that contain latency-associated peptides (LAPs) on their surface and act in vitro and in vivo via a TGF-β-dependent mechanism [97]. Although the expression of surface LAP may be induced in a Foxp3^+^-independent manner [98], both FoxP3 Treg and activated FoxP3 Treg have the ability to express LAP [99]. Therefore, the Foxp3+Treg and LAP Treg relationship is still unclear. The induction and inhibition of Foxp3^+^ Treg and LAP Treg are dependent on TGF-β. They both enable and produce IL-10, inhibiting the secretion of pro-inflammatory cytokines such as IFN-γ, IL-12, and TNF-α. Thus, there is some plasticity among different Treg subsets that exhibit the overlapping functions during immune regulation by oral antigen delivery [100]. Tr1 is a distinct subset of Treg, which mainly achieves the immunosuppression of inflammation by producing IL-10 and IL-21. It is worth noting that Foxp3^+^ Treg cells induced by TGF-β ‘instruct’ DCs to produce IL-27 and TGF-β, which supports the generation of IL-10-producing Tr1 cells [101], showing potential interactions between different Treg subsets.

#### 3.2.2. DCs

Immature T cells cannot respond to sensitizing proteins but are presented through APCs, resulting in an adaptive immune response or tolerance. The APCs are primarily involved in antigen presentation and include DCs and macrophages. DCs play a direct role in the mechanism of CD tolerance. At present, it is generally believed that there are two opposite pathways after DCs are stimulated by gluten: (1) A small number of migrated DCs expressing CD103 carry soluble antigens, migrate to the mesenteric lymph nodes (mLNs), and present antigens to naive T cells by relying on retinoic acid and TGF-β induction mechanisms. It promotes the differentiation of naive T cells into Treg tolerance [102]. Most immature DCs (iDCs) are rapidly converted to mature DCs (mDCs) after being stimulated by sensitized antigens and have intestinal homing characteristics, which promote the immune response and the infiltration of inflammatory cells. It is equally important for DCs to promote and inhibit the immune response. (2) After CD patients are exposed to the gluten antigen, DCs can express different costimulatory factors and adhesion factors according to the stimulation of gluten and its changes in tissue metastasis. Meanwhile, cytokines such as IL-6, IL-8, IL-10, TNF-α, MCP-1, and MCP-2, macrophage-derived chemokines, and an increase in RANTS secretion trigger a cytokine-rich microenvironment, accompanied by phosphorylation of p38 MAPK [103]. The changes in CD immune balance can be observed by monitoring the expression of DC costimulatory factors and human adhesion molecules. However, so far, it has been found that the costimulatory and adhesion factors cannot fully reflect the complex mechanism of metastasis and the maturation of DCs due to the sensitivity of DCs to the intestinal environment of patients with CD.

In comparison with DCs that promote immune responses, CD-related tolDCs are rarely reported. It was found by small intestinal biopsy that there was a close relationship between IDO^+^DCs and Foxp3^+^T cells. Different DC subsets were involved in the pathological process of CD, and tolDCs might be involved in the formation of Treg [104]. As shown in Figure 3b, it presents the relationship between CD and tolDCs and summarizes the tolerance mechanism of tolDCs in general [105]. (1) Programmed cell death ligand-1/2 (PDL-1/2) and cytotoxic T-lymphocyte-associated antigen-4 (CTLA-4) are highly expressed on the surface of tolDCs. (2) The tolDCs compete with CD80/CD86 to bind to T cells’ CD28 on the surface of other DCs. (3) The tolDCs release anti-inflammatory cytokines such as TGF-β, leading to the differentiation of Tregs that produce IL-10, and IL-10 inhibits the production of pro-inflammatory cytokines. (4) Indoleamine 2, 3-dioxygenase (IDO) and inducible nitric oxide synthase (iNOS) secreted by tolDC induce Treg [106]. It is worth mentioning that CTLA-4 was defective in CD patients at an early stage [107]. Therefore, unlike the general mechanism of tolDCs, part of the CD patient’s tolerance mechanism might lose function, resulting in an imbalance of tolerance. Initial cDCs and CD103^+^ DCs in lamina propria can promote immature T cells to differentiate into Treg, but the following still need to be elucidated: both the dose-effective relationship between tolDCs and antigen stimulation and the effect of CD on tolDCs in an inflammatory environment. 

#### 3.2.3. Goblet Cells

The mucous layer of the gastrointestinal tract is the front line of defense of the innate host. Its main component is the secretory mucin glycoprotein (MUC2) synthesized by intestinal goblet cells [108]. At the same time, goblet cells are also involved in the tolerance mechanisms of dietary antigens. Several modes of translocation of sensitizing antigens have been described for the intestinal epithelium. The goblet cells capture antigens differently from other pathways, which is associated with the induction tolerance sites of antigen-presenting cells in the lamina propria and the intestinal lumen. It has been found that goblet cell antigen channels (GAPs) are a mechanism by which oral antigens are transferred from the lumen to the extra basal space [109]. The goblet cells deliver antigens from the small intestine to the tolerant CD103^+^ DCs in the lamina propria. These cells cross-present antigens to T cells and induce tolerance [110]. In addition, the goblet cells promote tolerance to the existing Treg and layer DCs in the lamina propria, elevate the secretion of macrophages from the lamina propria IL-10, and impair tolerance to dietary antigens when goblet cells are lost [111]. Therefore, the potent oral tolerance induced by goblet cells may provide a new idea for CD to recover tolerance.

### 3.3. Reducing Gluten Diet Restrictions

Currently, it can often be challenging to obtain gluten-free products or specialized gluten-free meals and it may come with significant drawbacks. For instance, there can be nutritional imbalances in individuals sensitive to gluten. Therefore, the most ideal approach for treating CD is represented by the way of restoring tolerance to gluten products in CD patients. In 1988, Troncone and Ferguson [112] found that gliadin could inhibit the cellular and humoral immune responses to gliadin in mice when given gliadin or a gliadin-containing diet before gliadin sensitization, which proved that gliadin itself is an effective oral tolerance. Therefore, it can be considered to induce Tregs’ tolerance or deletion and anergy of T cells by oral administration of peptides containing T cell epitopes or trace gluten to achieve oral immune tolerance for the treatment of CD. In recent years, it has been found that the treatment method of modifying trace gluten to restore tolerance has strong maneuverability and rationality. This approach modifies the structure of the gluten to aggregate via the alteration of the amino acid charge, thereby shielding or reducing its allergenicity while retaining its ability to mediate tolerance. Freitag et al. [113] prepared negatively charged nanoparticles by encapsulated gliadin with poly(lactide), which induced the oral tolerance of CD in mice. To sum up, some methods were needed to restore the tolerance of gluten in patients with CD. Chemical deamidation, structural modification of physical methods, and intake of trace gluten may be effective ways to save costs and achieve tolerance recovery.

## 4. Research Progress on Dietary Polyphenols Used in CD Treatment

**Figure 4:**
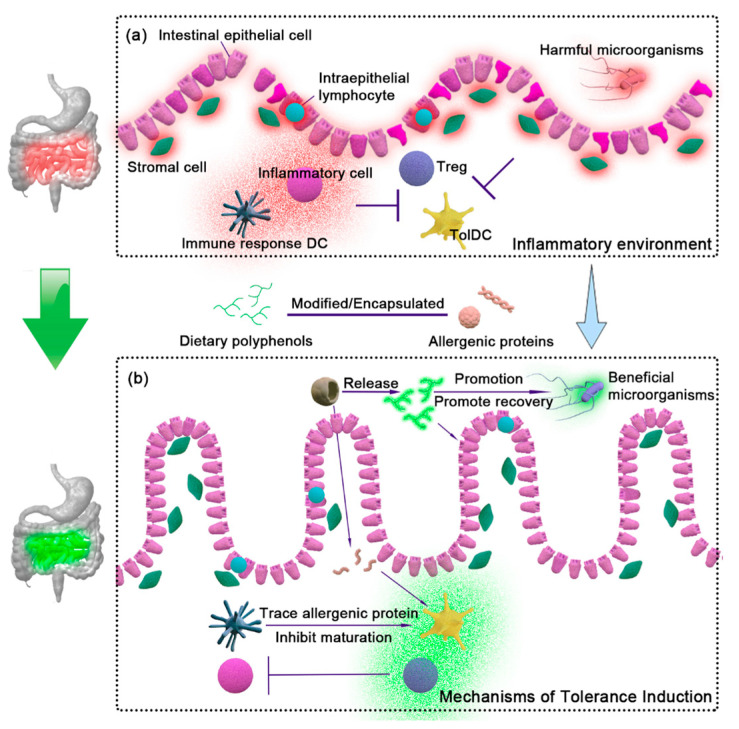
Hypothetical mechanism of polyphenol–gluten treatment of CD to restore oral tolerance. (**a**) Inflammation in the CD intestinal tract. (**b**) Polyphenol–gluten restores oral tolerance with CD. Wheat protein modifying or entrapping dietary polyphenols will release trace amounts of allergenic proteins after entering the intestinal tract, inhibit iDC maturation, promote T cell differentiation into Treg, and release dietary polyphenols to repair intestinal epithelial cells, regulate the intestinal flora, and restore oral tolerance in CD patients.

Dietary polyphenols are the secondary plant metabolites with a polyphenolic structure that are extensively present in everyday meals. Dietary polyphenols have a wide range of pharmacological and therapeutic properties as instant regulators, including inoxidizability, anti-inflammatory activities, and the inhibition of epithelial cell apoptosis. The mechanism of dietary polyphenols in the treatment of CD is divided into three parts: (1) Dietary polyphenols interact with sensitized gluten or peptides to change the structure of proteins or peptides. It causes their aggregation and precipitation, reducing the exposure of antigen epitopes, or changing the structure of antigen epitopes to a non-toxic structure. (2) Dietary polyphenols regulate the intestinal inflammatory environment, promote the proliferation of beneficial microflora florals, degrade toxic proteins or peptides, and downregulate the secretion of related inflammatory factors. They also affect epithelial inflammation-related cellular pathways and contribute to the repair of “intestinal leakage” caused by the disintegration of the Tight junction (Tj). (3) Dietary polyphenols control the immune response mediated by DCs and T cells, promote the formation of tolDCs and Treg, and clear the barrier to tolerance function. Therefore, given the mechanism of the action of dietary polyphenols in the treatment of CD, a hypothetical mechanism of polyphenol–gluten treatment of CD to restore oral tolerance is proposed in Figure 4.

### 4.1. Interaction Mechanism of Gluten and Dietary Polyphenol

**Figure 5:**
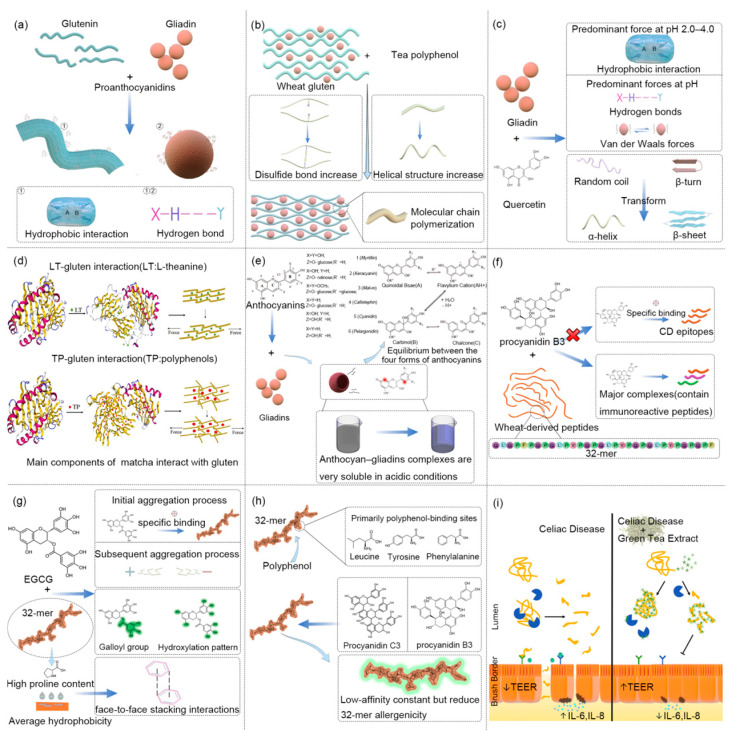
Interaction of gluten and dietary polyphenols. (**a**) Non-covalent linkage of proanthocyanidins to wheat proteins [115]. (**b**) Tea polyphenols promote structural changes and polymerization of wheat gluten [117]. (**c**) Different non-donor linkages between quercetin and gliadins at different pH and the structure of gliadin changes [118]. (**d**) Tea polyphenols form ternary hydrogen bonds with wheat protein to stabilize its network structure [116] (reproduced with permission from Ref. [116]. Copyright publisher Elsevier). (**e**) Anthocyans change the secondary structure of gliadin [119] (reproduced with permission from Ref. [119]. Copyright publisher Elsevier). (**f**–**h**) Interaction mechanism of proanthocyanidin B3, EGCG, green tea polyphenols, proanthocyanidin monomers, polymers, and wheat protein peptides [121,122] (reproduced with permission from Refs. [121,122]. Copyright publisher Elsevier). (**i**) The complexes of green tea polyphenols and gliadins regulate inflammation [123] (reproduced with permission from Ref. [123]. Copyright publisher Wiley Company).

Polyphenols interact with protein-digesting enzymes or substrates, exerting an anti-nutritional effect during protein metabolism. Studies began by exploring the structural characteristics and binding capacity of the interaction between gluten or a peptide and polyphenols in vitro. It was found that the harmful gluten or peptide sequestered in the digestive tract is prevented from being absorbed thanks to the anti-nutritional qualities of polyphenols. So dietary polyphenols are considered to have the effect of curing CD [114]. From a molecular point of view, the interaction between gluten and dietary polyphenols can be divided into covalent and non-covalent bonds, which include hydrogen bonds, electrostatic interactions, hydrophobic interactions, and van der Waals forces. Among them, the hydrogen bond and van der Waals forces are the main forces driving the binding of most polyphenols to gluten. The forms of non-covalent bonds have been widely studied because of the following merits: reversible actions, simple operation, and easy characterization. As shown in Figure 5, Girard et al. [115] reported that high-molecular-weight polymerized proanthocyanidins bound more closely to gluten through hydrophobic interaction and hydrogen bonds, and gliadin bound reversibly through hydrogen bonds. Liu et al. [116] discovered that tea polyphenols increased dough stability by forming a “grid” structure with a large number of intermolecular hydrogen bonds between gluten. Through the pasta system with a high content of green tea polyphenols, Han et al. [117] found that tea polyphenols enhanced the α-helix structure of the gluten, promoted the conversion of SH/SS, enhanced the hydrophobic interaction, and caused the polymerization of the gluten molecular chain. Most dietary polyphenols modify the structure of gluten, but their properties will not change. Instead, dietary polyphenols will be aggregated to improve their ability to make a difference due to the force of the gluten structure. Wang et al. [118] found that the transformation of a β-sheet and irregular roil to an α-helix might reduce the sensitization of gliadin in quercetin–gliadin interactions. Mazzaracchio et al. [119] pointed out that the secondary structure of the gliadins changed while anthocyanins did not change in the anthocyanin–gliadin complex. Dias et al. [120] observed that the complex might include immunoreactive peptides, and proanthocyanidin B3 exhibited a preference for binding to entropy-induced sites rather than specific ones. Subsequently, Dias et al. [121] indicated that epigallocatechin gallate (EGCG) showed high reactivity to 32mer. Here, the interaction mechanisms of self-assembly and aggregation are caused by the high overlap of proton signals. The above soluble complexes’ formation helped explain the binding site’s uncertainty. The high reactivity of EGCG and 33mer may be due to the following reasons: (1) EGCG may have some specificity at the beginning. However, the subsequent aggregation process seems to be dominated by the surface charge effect. (2) The average hydrophobicity and high proline content of 32mer provide a flat, rigid, and hydrophobic surface platform for EGCG, which causes the π–π accumulation of aromatic rings of EGCG to form stacks. (3) The hydroxylation of EGCG gallic acyl groups and aromatic rings cause strong interactions in the Caco-2 cell model. EGCG was able to significantly decrease the concentration of 32mer free peptides on the basal side of the monolayer. On this basis, Dias et al. [122] further reported that leucine, tyrosine, and phenylalanine were the main binding sites driven by energy in 32mer interactions with proanthocyanidins and green tea polyphenols. Although the results showed that proanthocyanidins did not bind closely to 32mer, the antigenic epitopes of 32mer were reduced. Van Buiten et al. [123] demonstrated that green tea polyphenols reduced the stimulation of gliadin on cell permeability and the release of IL-6 and IL-8, thereby regulating the inflammatory response of CD. It is proposed that the main mechanism of covalent bond formation is the production of free radicals from quinone. Quinone compounds further covalently react with a large number of nucleophilic side chains of lysine and cysteine, which will seriously affect the properties of individual proteins. However, this covalent interaction is not mature enough at the analytical level [124].

### 4.2. Effect of Dietary Polyphenols on Tolerance Mechanism

Dietary polyphenols exhibit significant anti-inflammatory and antioxidant effects and demonstrate the potential to modulate tolerance mechanisms in the immune system. Dietary polyphenols modulate immune responses and promote the establishment of immune tolerance by affecting the functions and signaling pathways of immune cells. Cong et al. [125] reported that curcumin-treated DCs induced intestinal immature CD4^+^ T cells to differentiate into Treg, including CD4^+^ CD25^+^ Foxp3^+^ Treg and Tr1. In another study of EGCG, Yoneyama et al. [126] found that EGCG induced the apoptosis of DCs in the differentiation stage and inhibited the endocytosis of iDCs while inhibiting mature DCs and stimulating allogeneic T cells. In addition, dietary polyphenols also affect the differentiation and the function of immune cells, and regulate the production and the release of inflammatory factors. The green tea polyphenols upregulated IL-10 and downregulated cytokines and enzymes such as IL-6, IL-8, IL-1β, TNF-α, NO, IL-17, PGE-2, COX-2, and so on [127]. Svajger et al. [128] discovered that resveratrol downregulated mature costimulatory factors, MHC-II, ILT3, and ILT4 in differentiated MoDCs. Meanwhile, it enhanced the ability of DCS to produce IL-10 and lost the ability to secrete IL-12 p70. It was worth mentioning that resveratrol played a role by interfering with DC differentiation of DCs and the translocation of NF-κB. Overall, dietary polyphenols positively or negatively regulate the functional activity of DCs by reducing inflammation or restoring immune function: (1) Dietary polyphenols downregulate specific signal pathways, induce the expression of tolerance-related molecules, and inhibit DC maturation. (2) Dietary polyphenols inhibit some transcription factors’ activity to counteract T cells’ ability to restrain and activate the immune response [129].

The study of dietary polyphenols and their effects on the immune system concerning CD is still in its basic stage. The molecular mechanism of dietary polyphenols in the human intestinal immune system remains to be elucidated. There are limited reports about using the CD model to study the mechanism by which polyphenols regulate tolerance. Therefore, it is helpful for the treatment of CD by drawing lessons from other autoimmune disease models. Wong et al. [130] discovered that EGCG could be used as a dietary DNA methyltransferase (DNMT) inhibitor to induce Foxp3 and IL-10 and to regulate the increase in Treg in the spleen and mLNs. De Santis et al. [131] observed that quercetin significantly increased the expression of the secretory leukocyte protease inhibitor (SLPI) in DCs. SLPI could inhibit the activation of NF-κB mediated by LPS, thus inhibiting the secretion of TNF-α. Campbell et al. [132] demonstrated that modulation of DC by sage and curcumin leads to inhibition of the mammalian target of rapamycin (mTOR) signaling in LPS-stimulated DCs through activation of AMP-activated protein kinase (AMPK). The secretion of heme oxygenase-1 (HO-1) by DC induced by these two polyphenols depends on the activation of AMPK [133]. Furthermore, Wang et al. [134] found that flavonoid naringin ligands induced CD4^+^ Foxp3^+^ Tregs through the aromatic hydrocarbon receptor (AhR) rather than the well-known TGF-β-mediated pathway. It is suggested that different dietary polyphenols may induce Tregs through different pathways.

### 4.3. The New Situation of the Interaction between Dietary Polyphenols and Gliadin

Dietary polyphenols are particularly sensitive to environmental conditions such as pH, salt, temperature, and light due to their water insolubility and significant antioxidant activity. Hence, it is not very good concerning their efficacy in treating diseases. With the rapid development of the food industry, amphiphilic proteins are used in solutions or emulsions containing active substances to form nano-encapsulated particles or micron emulsions, which can effectively improve the bioavailability and shelf life of active substances. Gliadin can be self-assembled into nanoparticles because its terminal region is usually more hydrophobic than the central region [135]. The application of gliadin can not only solve the problems of dietary polyphenols but also provide trace antigens for the tolerance treatment of CD. It should also be noted that the modified gliadin could exert a better protective effect on this basis (Table 2).

Many studies have focused on the formation of nano-encapsulated particles between gliadin and dietary polyphenols. Liu et al. [136] discussed that nanoparticles prepared from gliadin and proanthocyanidins (PACs) had significantly higher antioxidant activity than pure PAC. Guo et al. [137] reported that the nanoparticles formed by encapsulating curcumin with gliadin had good stability and improved the bioavailability of curcumin. Wu et al. [138] showed that the synthesized resveratrol-loaded nanoparticles with gliadin had the characteristics of higher encapsulation efficiency and stability. In summary, these studies demonstrate that gluten-based nanoparticles significantly enhance the antioxidant activity, the bioavailability, and the stability of dietary polyphenols, providing a theoretical foundation for the treatment of CD. Many studies have also been conducted using micron-sized emulsions to deliver dietary polyphenols. Ma et al. [139] reported that the Pickering emulsion prepared with β-carotene and gliadin-gum Arabic had a storage modulus to transport β-carotene. Fu et al. [140] found that Pickering emulsions prepared by encapsulating β-carotene in gluten or gluten–xanthan gum effectively prevented the chemical degradation of β-carotene. Chen et al. [141] improved the chemical stability of EGCG, the solubility of quercetin, and the bioavailability of both by encapsulating EGCG and quercetin in the W/O/W emulsion gel system. However, most studies measuring the bioavailability of dietary polyphenols have only involved data on the polyphenols released from the food substrates that protected them or their subsequent concentrations in urine [129]. The plasma concentration of dietary polyphenols should be more appropriate as a sign of the release and absorption of dietary polyphenols from the food matrix. The most commonly used method in human research is a single dose, which means that it is temporary toward the increase in the concentration of polyphenols in the blood. It mainly reflects the ability of the body to absorb dietary polyphenols from food, but cannot represent the index of physiological concentration. Therefore, more attention should be paid to the replacement of accurate bioavailability indicators based on the protection of dietary polyphenols.

## 5. Conclusions and Future Perspective

Gluten occupies a significant part of the wheat raw material and forms the gluten network through the process of hydration and mixing. This unique structure confers the ability to process gluten into a wide variety of food products. Currently, a number of substantial advances have been made in the study of the gluten network in terms of its supportive and rheological properties in the dough. However, gluten networks undergo structural changes during subsequent processing that give them different roles in food products. For example, gluten networks swell during fermentation to retain gas, thereby affecting the fluffiness of bread. Therefore, it is still necessary to conduct in-depth studies on the role of gluten in various types of foods after processing.

Meanwhile, gluten is a complex protein whose digestion in living organisms is crucial for providing nutritional advice. The digestibility of gluten is affected in complex food matrices. Researchers can improve the bioavailability of gluten by studying the digestibility of gluten in different factors. From a production perspective, it will contribute to a shift in gluten processing from the current empirical approach to more rational and specific processing principles.

In addition, the unique gluten structure rich in glutamine and proline repeat sequences provides important information for the perception and diagnosis of celiac disease. Researchers are actively seeking methods to alleviate dietary restrictions related to gluten. It is worth noting that many studies have discovered that CD103^+^ DC subsets of DC and Tr1 and Foxp3^+^ Treg subsets of Treg play an important role in the recovery of CD tolerance, which defines the research goals for future tolerance work. There has also been noteworthy progress in the study of how different dietary polyphenols modulate the intestinal immune system and gluten encapsulation technology. The use of gluten-encapsulated/modified dietary polyphenols to restore CD tolerance and reduce the need for gluten dietary restriction is a highly promising approach, based on understanding the interaction between gluten and dietary polyphenols. Overall, research on gluten faces multiple challenges, including the properties of gluten, the chemistry during processing, and its role in different processing. Effective control and manipulation of gluten can reduce its dietary restriction. It is an important issue that cannot be ignored in future food processing.

## Figures and Tables

**Table 1:** Factors influencing the digestibility of gluten.

	Factors	Digestibility of Gluten	Mechanism of Influence	References
Internal	The amino acid sequence of the proteins	The higher the proline content, the lower the digestibility	Gluten is rich in proline, making it difficult for enzymes to break down.	[73]
	Protein folding and cross-linking	Reduce digestion	Tight protein folding or protein aggregation limits enzyme cleavage sites and affects gluten digestibility.	[74]
External	Protease inhibitors	Reduce digestion	Protease inhibitors decrease protein digestibility by inactivating digestive proteases.	[75]
	Starch	Improve digestion	Starch protects gluten from aggregation in water, disrupts the spatial structure of gluten, exposes more cleavage sites, and facilitates gluten digestion.	[76]
	Tannin	Reduce digestion	Tannins reduce gluten digestibility by denaturizing proteases, inhibiting intestinal amino acid transporters, and complex glutens.	[77]
	Dietary fiber	Reduce digestion	Dietary fiber surrounds gluten, creates a steric hindrance between gluten and proteases, and compresses gluten conformation, inhibiting proteolysis by proteases.	[78]
	Low pH	Improve digestion	Acidic deamidation of gluten occurs at low pH and is accompanied by partial hydrolysis of peptide bonds.	[79]
Processing	Grind	Improve digestion	Cellular structures are split in grinding and the gluten matrix is exposed to the environment and hydrolases.	[68]
	Shear	Unchanged	-	[80]
	Heat	Reduce digestion	Heating changes the degree of network interconnection within the thiol-rich gliadin and thus the structure of gluten in bread.	[81]
	Extrusion	Improve digestion	Extrusion treatment increases the structural flexibility of wheat proteins and exposes more restriction sites.	[82]
	Fermentation	Improve digestion	Gas production and capture during fermentation maximize the separation of parallel protein chains and limit gluten cross-linking during baking.	[83]
	Cold atmospheric plasma	Improve digestion	Generating numerous high-energy excited atoms, photons, electrons, and reactive oxygen and nitrogen species modifies gluten to depolymerize gluten proteins, reducing the amount of large-sized protein polymers and decreasing immunoreactivity.	[84]

**Table 2:** Protein-based nano-encapsulation or micron emulsions improve the efficacy of dietary polyphenols by overcoming solubility difficulties. Gliadin acts as a carrier for CD tolerance therapy, with the benefits of enhancing its protective benefits.

System	Polyphenol	Particle Size	Activity (Details of Research)	References
Nano-encapsulated particles	Proanthocyanidins	Around 30 nm	No cytotoxicity for normal liver cells Exhibited clear cytotoxicity against liver hepatocellular carcinoma	[122]
	Curcumin	196.66 nm	Increase bioavailability	[123]
	Resveratrol	Around 300 nm	Improve bioavailability Improve chemical stability Improve dissolution Improve antioxidant activity	[124]
Micron emulsions	β-carotene	251.3 ± 5.1 nm	Improve stability No effect on lipid digestion or carotenoid bioaccessibility	[125]
		Wheat gluten nanoparticle–xanthan gum: 23.9 μm; wheat gluten nanoparticles: 9.4 μm	Effective protection from chemical degradation Increase bioavailability	[126]
	Quercetin	-	Enhance solubility Bioavailability increased 4 times	[127]
	EGCG		Improve chemical stability Bioavailability increased 2 times	

## Data Availability

Not applicable.
